# Pregnancy as a susceptible state for thrombotic microangiopathies

**DOI:** 10.3389/fmed.2024.1343060

**Published:** 2024-02-27

**Authors:** Marie Frimat, Viviane Gnemmi, Morgane Stichelbout, François Provôt, Fadi Fakhouri

**Affiliations:** ^1^CHU Lille, Nephrology Department, Univ. Lille, Lille, France; ^2^Inserm, Institut Pasteur de Lille, Univ. Lille, Lille, France; ^3^Pathology Department, Univ. Lille, Lille, France; ^4^Service of Nephrology and Hypertension, CHUV and University of Lausanne, Lausanne, Switzerland

**Keywords:** women, pregnancy, endothelium, thrombotic microangiopathies, complement activation, angiogenesis factors, kidney biopsy, placental histology

## Abstract

Pregnancy and the postpartum period represent phases of heightened vulnerability to thrombotic microangiopathies (TMAs), as evidenced by distinct patterns of pregnancy-specific TMAs (e.g., preeclampsia, HELLP syndrome), as well as a higher incidence of nonspecific TMAs, such as thrombotic thrombocytopenic purpura or hemolytic uremic syndrome, during pregnancy. Significant strides have been taken in understanding the underlying mechanisms of these disorders in the past 40 years. This progress has involved the identification of pivotal factors contributing to TMAs, such as the complement system, ADAMTS13, and the soluble VEGF receptor Flt1. Regardless of the specific causal factor (which is not generally unique in relation to the usual multifactorial origin of TMAs), the endothelial cell stands as a central player in the pathophysiology of TMAs. Pregnancy has a major impact on the physiology of the endothelium. Besides to the development of placenta and its vascular consequences, pregnancy modifies the characteristics of the women’s microvascular endothelium and tends to render it more prone to thrombosis. This review aims to delineate the distinct features of pregnancy-related TMAs and explore the contributing mechanisms that lead to this increased susceptibility, particularly influenced by the “gravid endothelium.” Furthermore, we will discuss the potential contribution of histopathological studies in facilitating the etiological diagnosis of pregnancy-related TMAs.

## 1 Introduction

The terms “thrombotic microangiopathy” (TMA) or “TMA syndrome” (1, 2) encompasses a diverse array of clinical entities sharing a common histological presentation characterized by endothelial cell alterations and frequent fibrino-platelet thrombi restricted to the microvasculature (3). The biological manifestations of TMA are typically characterized by the concurrent presence of consumptive thrombocytopenia and microangiopathic hemolytic anemia (MAHA), both of which serve as reliable indicators alerting clinicians to the possibility of a TMA syndrome. The presence of one or more organ predominant dysfunctions resulting from the downstream ischemic effects of microvascular damage, has historically been used to distinguish the different TMA syndromes: predominant neurological impairment in thrombotic thrombocytopenic purpura (TTP), acute renal failure in hemolytic uremic syndrome (HUS), or hepatic cytolysis in the context of HELLP (Hemolysis, Elevated Liver enzymes, Low Platelet count) syndrome in pregnant women (4, 5). This clinical categorization is susceptible to fallibility due to the presence of intragroup heterogeneity and potential overlap between distinct syndromes, thereby challenging precise differentiation (6–8).

The last 40 years have witnessed substantial advancements in elucidating the intricate mechanisms underlying TMAs (2, 9). In the 1980s, the discovery of a deficiency in a von Willebrand factor-cleaving protease, ADAMTS13 (a disintegrin and metalloproteinase with a thrombospondin type1 motif– member 13), provided a pivotal breakthrough (10, 11). This deficiency was identified as a key contributor to the accumulation of unusually large von Willebrand factor multimers, leading to platelet aggregation and microthrombi formation characteristic of TTP (12, 13). Another series of significant advances emerged in the early 2000s, centered on the identification of abnormalities - genetic variants or acquired autoantibodies - affecting the regulation of the complement alternative pathway in patients with atypical HUS (aHUS) (14–16). This has emphasized the significance of disrupted regulation of complement, particularly via uncontrolled activation of the alternative C3 convertase and the ensuing stimulation of C5 convertase. Ultimately, the release of the C5a anaphylatoxin and most importantly of the C5b-9 terminal complement complex leads to the development/progression of endothelial lesions and thrombi (17, 18). Additional causal or associated factors of TMA (e.g., infection, medication, etc.) have also been identified with varying degrees of understanding of the mechanisms involved (2, 9, 19). These findings have enabled a more refined categorization of TMA subtypes based on their distinct etiological factors rather than relying solely on syndromic presentations. Consequently, the diagnostic and categorization of TMA now aspire to be grounded in physiopathological mechanisms, as outlined by the KDIGO guidelines (20).

This conceptual framework has forged pathways for the implementation of targeted therapeutic modalities, as exemplified by the utilization of plasma exchanges combined with or without anti-CD20 treatment in TMAs characterized by autoantibody involvement (e.g., cases of anti-factor H antibody-associated aHUS, ticlodipine-induced HUS, and immune-related TTP). Additionally, the deployment of C5 blockers in complement-mediated HUS (CM-HUS), and more recently, the integration of caplacizumab in the therapeutic armamentarium for TTP, attests to the precision and efficacy afforded by this paradigm. However, there are still many challenges. It is important to note that the mechanisms for most TMA cases are multifactorial and complex, with interactions between these factors contributing to the development and progression of the condition. Unraveling the intricate interplay between these factors and their roles in transitioning susceptibility to pathology could hold promise for the identification of diagnostic and follow-up markers, which are notably deficient apart from TTP, as well as for the development of additional targeted therapeutic strategies.

This review will focus on the specific context of pregnancy and the postpartum period, conditions that exemplify the inherent complexity of TMA pathophysiology and the challenges of deciphering the predominant causal factor. During pregnancy, intricate physiological adaptations occur within the vascular milieu, rendering the endothelial lining more vulnerable to disruptions in hemostasis. While the exact mechanisms are not fully elucidated for all TMA syndrome, this heightened susceptibility is attributed to a multifaceted interplay of hormonal, immunological, coagulation and hemodynamic factors, culminating in an environment conducive to dysregulated endothelial activation and subsequent TMA development, particularly in case of additional diseases or risk factors.

This review will examine the characteristics of TMA during pregnancy, refraining from detailed one-by-one descriptions and excluding discussions on management, as these have recently been comprehensively addressed (4, 5, 21). Instead, we will emphasize available tools for distinguishing pregnancy-related TMAs, with special attention to the potential contributions of histopathological analysis. Furthermore, this review aims to decipher the underlying mechanisms that promote susceptibility to TMA lesions during pregnancy and drive the transition from predisposition to pathological manifestations, with a particular focus on the influence of the “gravid endothelium.”

## 2 Differentiating and diagnosing pregnancy related-TMAs

### 2.1 What TMA syndromes?

Pregnancy and the postpartum period, usually spanning up to 3 months post-delivery, are well-established significant risk factors for TMA (4, 5). TMA can manifest in diverse conditions, including exclusive pregnancy-specific patterns referred to as “pregnancy-specific TMAs” like preeclampsia (PE) and HELLP syndrome, and patterns associated with specific diseases or conditions, collectively termed “pregnancy-associated TMAs,” the incidence of which rises within the pregnancy context (4, 5). Pregnancy-associated TMAs encompass TTP, CM-HUS, autoimmune disorders such as systemic lupus erythematosus and antiphospholipid syndrome (APS), notably the catastrophic APS (CAPS). Despite suggestions, it remains unconfirmed whether pregnant women with severe diffuse scleroderma have a higher incidence of renal crisis, a TMA-like disorder, compared to non-pregnant women (22, 23).

Moreover, both PE and HELLP syndrome may coincide or emerge as a result of pregnancy-associated TMAs, adding to the intricacy of diagnosis and management (24–27). Further complicating matters, TMA-like patterns may also be noted in case of obstetrical disseminated intravascular coagulation (DIC). This severe event, characterized by extensive thrombus formation (not limited to small vessels), is associated with various pregnancy complications, including acute peripartum hemorrhage, placental abruption, sepsis, PE/HELLP syndrome, retained stillbirth, amniotic fluid embolism, and acute fatty liver of pregnancy (28–30). Hence, any suspicion of TMA should prompt repeated analysis of platelet count, coagulation parameters (such as prothrombin time and fibrinogen and fibrin split products/D-dimer concentrations), while considering the pregnancy-associated intricacies of these metrics. This consideration has prompted certain researchers to advocate the use of pregnancy-specific diagnostic scoring systems for obstetrical DIC including fibrinogen concentrations, the PT difference and platelet count (31).

The biological criteria for TMA in pregnancy closely resemble those in the general population, except for the lower threshold of thrombocytopenia. Indeed, in the context of a healthy pregnancy, platelet count moderately decreases, gradually starting from the second trimester, reaching a nadir in the third trimester, while still remaining above 80 × 10^9^/L. This benign gestational thrombocytopenia has been related to hemodilution due to increased plasma volume, augmented platelet consumption in the utero-placental unit and potential heightened platelet clearance, and does not affect pregnancy outcome (32, 33). Diagnosis of pregnancy-related TMA is thus based on the following parameters: platelet count <100 × 10^9^/L, hemoglobin level < 10 g/dL, serum lactate dehydrogenase >1.5 times the upper limit of normal, undetectable serum haptoglobin, negative direct erythrocyte antiglobulin test, and presence of schistocytes on blood smear (5).

### 2.2 How do pregnancy-related TMAs differ?

The detection of MAHA in a pregnant or newly delivered patient constitutes a diagnostic and therapeutic emergency. In this context, algorithms for the management of pregnancy-related TMA are regularly updated, considering the challenge of urgent differentiation (5, 21, 34). Table 1 provides a concise overview of pregnancy-specific and -associated TMAs, focusing on the distinguishing features of different forms of TMA.

**Table 1:** Main features of pregnancy-specific TMAs and pregnancy-associated TMAs.

TMA syndromes in pregnancy	Epidemiology	Key-elements for diagnosis			Physiopathology	Treatment modalities
		Context/timing	Most discriminating clinico-biological symptoms	Impact of delivery		
PE with renal dysfunction	1.7% of all HDP 15% of all PE	From 20 weeks gestation to 4 weeks PP	new-onset HT andAKI ^Δ^ Ratio of sFLT1/PlGF > 85	Improvement in 48-72 h PP. Proteinuria may persist >1 year	Abnormal placentation with release of antiangiogenic markers, mediated primarily by sFlt-1 and sEng	Delivery, supportive care
HELLP	0.2–0.6% of all pregnancy	Previous PE (80% of HELLP)/T3 to early PP	Elevated liver enzymes AST or ALT > 70 IU/L	Improvement in 48-72 h PP	Few understood: Abnormal placentation (continuum with PE); Complement AP dysregulation; DIC	Delivery, supportive care
TTP	5.10^−3^ to 10^−4^% of all pregnancy	TTP history/iTTP: T3, early PP; cTTP: from T1	ADAMTS-13 < 10% platelets < 30.10^9^/L	No	Congenital or acquired ADAMTS-13 deficiency leading to accumulation of large VWF multimers	PEX, caplacizumab*, IS (iTTP), plasma infusion (cTTP)
CM-HUS	4.10^−3^% of all pregnancy	HUS history/from T3 to 3-months PP (80%)	Severe AKI Congenital or acquired abnormalities of complement system	No	Dysregulated activation of complement alternative pathway	PEX, C5 blocker
CAPS	1% of all APS/8% of all CAPS	Context of known APS or criteria +/within 4 weeks PP (80%)	± anti-cardiolipin ± anti-β2GPI antibodies ±lupus anticoagulant	No	Few understood: direct endothelial injury by aPL; dysregulated complement activation	Anticoagulation, PEX, IS
SLE	TMA in 17–24% of all lupus nephritis	Context of known SLE or LN	± Positive antinuclear ± anti-native DNA (anti-Ro/SSA)	No	Few understood: ADAMTS-13 activity deficiency (TTP-like syndrome); dysregulated complement activation; secondary APS	IS
DIC	0.03 to 0.35% of all pregnancy	Context of obstetrical complications	DIC (prolonged PT, thrombocytopenia, low fibrinogen)	No	DIC activates coagulation and triggers fibrinolysis	Cause-based treatment, supportive care

AID, auto-immune diseases; APS, antiphospholipid syndrome; AST, aspartate aminotransferase; ALT, alanine aminotransferase; GIT, gastrointestinal tractus; HT, hypertension; HDP, Hypertensive disorders of pregnancy; IS, immunosuppression; TI, topoisomerase; PEX, plasma exchange; PlGF, placental growth factor; PP, postpartum; SLE, Systemic lupus erythematosus; SRC, Scleroderma renal crisis; T1, first trimester of pregnancy; T3, third trimester of pregnancy.

^Δ^The definition used in these reports is based on a serum creatinine 0.90 mmol/L and/or a 0.25% increase compared with baseline values.

^*^caplacizumab is not approved for ongoing pregnancy (small molecular size, transplacental transfer). Nevertheless, it has shown success in pregnant women with acquired TTP, resulting in favorable outcomes (35, 36).

#### 2.2.1 Context and timing

Though non-specific, the context (e.g., significant personal/family history, occurrence in conjunction with PE or postpartum hemorrhage) and the timing of TMA onset during pregnancy provide informative insights. PE, the most common cause of TMA during pregnancy, is diagnosed through the identification of new-onset hypertension after 20 weeks’ gestation, concomitant with proteinuria and/or acute organ dysfunction (e.g., acute kidney injury (AKI), liver dysfunction, neurological features, hemolysis or thrombocytopenia, or fetal growth restriction) (37, 38). Although it is widely presumed that diverse subtypes of PE may exist, each likely driven by distinct pathophysiological mechanisms (39), there is no clearly delineated association between TMA features and a specific subtype of PE, particularly concerning its early or late onset. TMA is notably linked to severe forms of PE, especially the patterns associated with renal dysfunction and AKI (40–43). The distinction between PE complicated by TMA and HELLP syndrome remains subtle, especially in cases where HELLP complicates PE, and it is possible that a continuum exists between these conditions (4, 44, 45).

TTP and CM-HUS are rarer (occurring in approximately 1 in 20,000 to 200,000 pregnancies), but timely recognition is critical for improving maternal and fetal outcomes through targeted interventions. Pregnancy is a common circumstance for initial diagnosis or relapse of these diseases, unveiling ~20% of TTP and CM-HUS in childbearing-age women (46–51). While TTP is more common in the third trimester and early postpartum period (27), it can emerge as early as the first trimester, especially in case of congenital TTP (52, 53). The characterization of pregnancy-related HUS is even more limited; however, it predominantly arises during the postpartum period, with a notable 80% of cases occurring between the immediate post-delivery phase and the fourth postpartum month in a European cohort (47), and 73% in a Spanish cohort (54).

While a history of autoimmune disease contributes to understanding causative factors, pregnancy can also serve as a trigger for these pathologies first diagnosed during pregnancy. Pregnancy-associated TMAs are observed in systemic lupus erythematosus, particularly involving anti-SSA/Ro antibodies associated with renal TMA in lupus nephritis (24, 55). Approximately 4–6% of CAPS occur during the third trimester of pregnancy or postpartum, and half of the patients had a history of APS (56–58).

#### 2.2.2 Initial presentation

The first clinical and biological evaluation also lacks decisive factors but includes certain indicators. Blood pressure typically exceeds 140/90 mm Hg in PE/HELLP syndrome and CM-HUS cases, while it usually remains normal in TTP and is low in situations associated with obstetric severe complications and/or DIC. A recent study suggests that serum creatinine levels ≥1.9 mg/dL, LDH levels ≥1,832 units/L, or a combination of LDH levels ≥600 units/L and serum creatinine level ≥ 1.9 mg/dL can effectively differentiate CM-HUS from HELLP syndrome in the postpartum period (59). Furthermore, it has been suggested that a high LDH to AST ratio > 22 may indicate a higher likelihood of TTP rather than HELLP syndrome in a third-trimester pregnant patient suspected of having TMA (60, 61).

Renal failure may occur in all patterns of pregnancy-related TMAs, but a rapidly evolving AKI is more indicative of HUS or obstetric severe complications. Oligo-anuria may precede an increase in creatinine levels and serve as an early indicator of the extent of renal damage. Anuria may be a sign of renal cortical necrosis, a potential complication in both HUS and severe peripartum conditions, especially when linked to DIC in the latter case (62, 63). AKI is less common and relatively mild in TTP, but thrombocytopenia is more severe. The utilization of the PLASMIC score and the French score, developed to predict ADAMTS13 deficiency, can be helpful, although they have not been validated in pregnancy and may prove less discriminative in the setting of pregnancy (64). Renal involvement can be observed in both PE and HELLP syndrome. In a recent study, AKI was even more prevalent in HELLP patients than in those with preeclampsia (14.4% vs. 4.7%), likely due to higher rates of placental abruption or postpartum hemorrhage in HELLP syndrome (65). Acute tubular necrosis was the predominant finding in persistent AKI with HELLP syndrome, but coexisting TMA lesions are not uncommon (4/16 biopsies) (66). Liver function tests are likely to offer more discriminative diagnostic insights. While elevated liver enzyme levels serve as diagnostic criteria for both PE and HELLP syndrome (67, 68), they are considered a requisite for the diagnosis of HELLP syndrome according to the Mississippi classification (68). Moreover, the elevation of liver enzymes is notably more pronounced (6–8 times) in cases of HELLP syndrome compared to severe PE (69, 70).

#### 2.2.3 Outcome

The progression over the initial 72 h can also retrospectively assist in distinguishing pregnancy-related TMA (5). Spontaneous improvement in hemolysis and thrombocytopenia following delivery is common in cases of PE or HELLP syndrome, in contrast to other pregnancy-related TMAs. A retrospective study that examined the dynamics of various biological parameters in 105 patients with immediate post-partum AKI found that changes in hemoglobin, haptoglobin, and liver enzymes were not effective discriminators. Conversely, the evolution of platelet count showed statistically significant differences between primary TMA-related AKI and other groups, including AKI associated with PE/HELLP syndrome or post-partum hemorrhage (71).

### 2.3 Are there any specific biomarkers?

The recommended initial workup for pregnant or postpartum women presenting with TMA is detailed in recent reviews (5, 21). This workup includes coagulation tests, quantification of ADAMTS-13 activity, investigation of autoimmune disorders (lupus anticoagulant, anticardiolipin antibodies, Beta-2-glycoprotein antibodies, antinuclear antibody) or metabolite deficiencies (vitamin B9, B12), exploration of complement levels (C3, C4, factor H, factor H autoantibody, factor I, factor B, and MCP/CD46 expression by flow cytometry), and, when available, measurement of sFLT1 and PlGF concentrations.

The most specific indicator is ADAMTS13 activity. An activity of less than 10%, definitively confirms TTP, while levels above 20% usually rule out TTP. Hence, it should be urgently assessed in all cases of pregnancy-MAHA. In contrast, there is currently a lack of specific biomarkers for distinguishing between CM-HUS, PE, or the HELLP syndrome.

#### 2.3.1 Complement system

Quantitative levels of complement regulatory proteins lack both sensitivity and specificity to distinguish CM-HUS from other forms of pregnancy-TMA. Normal test results do not exclude the possibility of CM-HUS. During the acute phase, serum levels of C3, C5a, and sC5b-9 were within the normal range in ~50% of CM-HUS patients in two large series (72, 73). More recently, soluble C5b-9 levels were found elevated in only 21% of CM-HUS patients (74). The sensitivity of factor Ba exhibited better performance, with elevated levels found in 95% of CM-HUS cases. This study also identified urine Ba and soluble C5b-9 as potential biomarkers for CM-TMA, as both consistently display elevated levels in adults with aHUS in comparison to the highest levels observed in normal donors (74). There is currently no data available regarding these urinary biomarkers in the pregnant population.

Changes in plasma concentrations of complement proteins, including an elevation in complement activation products, have been documented in various types of pregnancy-related TMAs (27, 75, 76). Plasma levels of C3a, C4d, and sC5b-9 were 1.8-fold, 1.5-fold, and 1.2-fold higher in preeclamptic patients compared to healthy pregnant women (76). Elevated detection of complement activation products during pregnancy-related TMAs has been corroborated in other cohorts (77, 78) with some showing complement activation occurring as early as the first trimester (79).

Notably, pregnant women with elevated plasma factor Bb levels in early pregnancy had a heightened risk of later developing preeclampsia (80). In a study comparing the proteomic profiles in sera of patients who subsequently developed early-onset PE versus those with normal pregnancies, factor B was identified among the 12 proteins that showed differential expression (down-regulated with fold changes of −0.24) (81). In a similar approach, using blood samples collected immediately after the confirmation of PE diagnosis, the primary differential pathway identified in early-onset severe preeclampsia was related to complement and coagulation (82). In the PROMISS cohort, including 487 pregnant women with SLE or APS, elevated complement activation products (Bb and sC5b-9) detected as early as 12–15 weeks into pregnancy were significantly associated with adverse pregnancy outcomes (fetal/neonatal death, preterm delivery <36 weeks because of placental insufficiency or preeclampsia and/or growth restriction <5th percentile). This association remained strong even after adjusting for demographic and clinical risk factors, with a particularly robust link observed in patients with APS (75).

Elevated plasma levels of sC5b-9 and factor Bb have also been documented in cases of severe delayed postpartum hemorrhage (83), as a potential consequence of significant endothelial stress. Other tests, less commonly used in clinical practice, have also demonstrated complement activation in other TMAs besides CM-HUS. Through the application of an *ex vivo* assay assessing C5b-9 formation on endothelial cells induced by patients’ serum, enhanced C5b-9 deposition was observed in samples obtained from individuals with HELLP syndrome and preeclampsia (82, 84). This enhanced *ex vivo* C5b-9 deposition persisted up to 3 months postpartum, when patients were in clinical remission (84). Moreover, increased positivity of the modified Ham test was also noted in HELLP syndrome patients compared to those with normal pregnancies (85).

#### 2.3.2 Angiogenesis factors

The imbalance between placenta-derived anti-angiogenic factors, notably the soluble fms-Like Tyrosine Kinase 1 (sFLT1) and soluble endoglin (sEnd), and angiogenic proteins as placental growth factor (PlGF), represents a critical event in the pathogenesis of both PE (86, 87) and HELLP syndrome (88). Thus, the measurement of these angiogenic and anti-angiogenic factors is increasingly used for the prediction or early diagnosis of these disorders. An sFlt-1/PlGF ratio above 85 before 34 weeks and 110 thereafter indicates possible PE or HELLP syndrome. In contrast, ratios below 38 suggest an alternative diagnosis with a high negative predictive value (89, 90). In a cohort of 1,117 patients presenting with PE symptoms, the median sFlt-1/PlGF level among those experiencing adverse outcomes, whether maternal (AKI, HELLP syndrome, pulmonary edema, DIC, cerebral hemorrhage, or eclampsia) or fetal, was significantly higher compared to patients without adverse outcomes (median 177 [IQR, 54–362] versus 14 [IQR, 4–62]). This suggests that incorporating the sFlt-1/PlGF ratio into the diagnostic process could aid in detecting adverse outcomes in women suspected of having preeclampsia (91).

Elevated sEnd levels were also detected in early-onset PE and HELLP syndrome, showing a strong correlation with serum levels of sFlt-1/PlGF (92, 93). Therefore, mean sEnd levels were elevated at delivery in 72 pathological pregnancies (67 pg./mL in PE and 76 pg./mL in HELLP), compared to 10 normal pregnancies (5 pg./mL), but there was no statistically significant difference between PE and HELLP. These findings pertain to HELLP syndrome in conjunction with preexisting PE, not isolated HELLP syndrome (94). In a study comparing sFlt-1/PlGF ratios among isolated PE, PE/HELLP, and isolated HELLP syndrome, the ratios significantly varied between the three groups. The highest levels were observed in the PE/HELLP group (287 [51–948]), whereas cases with isolated HELLP syndrome exhibited the lowest ratios (49 [3–405]) (95). PE/HELLP and isolated HELLP syndrome exhibit distinct angiogenic behaviors, suggesting that they are two distinct entities.

No direct association between an increase of angiogenic factors and the occurrence of pregnancy related-AKI or -TMAs has been documented. Recently, the endothelial cell-derived factor DEL-1 has been suggested as a potential diagnostic tool for HELLP syndrome in pregnancy-TMA, this factor being decreased in HELLP as compared to PE. Further prospective studies are needed for confirmation (96). Other emerging biomarkers have been identified, but no associations have been reported with PE associated with AKI or TMA (97). Few studies have investigated the levels of placental-derived angiogenic factors in other non-specific pregnancy-related TMAs. Some data have emerged in the context of TTP (98).

## 3 Does histopathological analysis offer insights into distinguishing pregnancy-related TMAs?

During the acute phase of a pregnancy-related TMA, conducting a biopsy of the affected organ is not a standard procedure, primarily due to the associated risk of hemorrhage. Thus, histological analysis cannot be utilized to guide the diagnosis and immediate therapeutic intervention. Nevertheless, it is valuable to summarize the limited available data to identify potential pathological variations based on the underlying cause of pregnancy-TMAs. The histological features of pregnancy-related TMAs are outlined in Table 2. Another aspect that will be discussed in this section is the potential relevance of placental histology. In cases of immediate postpartum TMAs, the placenta is accessible, and we will assess whether it can serve as a tool for distinguishing various patterns of pregnancy-related TMA.

**Table 2:** Histological features of pregnancy-specific TMAs and pregnancy-associated TMAs.

TMA syndromes in pregnancy	Target organ of **TMA**	Characteristics of TMA lesions	
		Morphologic changes/location	Preponderant mechanisms of capillaries obstruction
PE	Kidney +++ (rare in others organs except in eclampsia patients (25%) or in necropsy: liver, heart, adrenal)	Endothelial vacuolization, hypertrophy of the cytoplasmic organelles. Thrombi rich in fibrin are infrequent (severe disease). No vasculitis/Primary glomerular lesions, rare in arterioles	Swelling of EC and accumulation of subendothelial electrodense deposits
HELLP	Liver +++ Kidney	Fibrin deposition with periportal hemorrhage and hepatocyte necrosis/Sinusoids and portal tract capillaries, hepatic arteries, ± portal vein branches	Vasospasm, intraluminal fibrin and swelling of EC
TTP	CNS +++ Described in kidneys, adrenals, heart, liver, spleen. Few in GIT. Classically not in the lungs	Thrombi mainly consisted of aggregated platelets, relative absence of fibrin. No vasculitis, no fibrinoid necrosis/Diffuse in brain; Glomeruli, arterioles, and interlobular terminal arteries in kidney	Platelets thrombi rich in vWF and factor VIII Swelling of EC
CM-HUS	Kidney +++ Frequent: Colon, pancreas, myocardium, CNS Possible: adrenals, skin and lungs	EC swelling, subendothelial deposition of fibrinoid, thrombi rich in platelets with presence of fibrin. No vasculitis/Glomerular capillaries, afferent arterioles ± intralobular arteries	Platelets thrombi Swelling of EC
CAPS	Kidney lung, CNS, heart, skin, liver, GIT	Microthrombi composed of fibrin, acute necrotizing vasculitis. In chronic cases, these lesions can be accompanied by/small arteries and arterioles	Fibrin thrombi Intimal proliferation, EC swelling + frequent fibrous intimal hyperplasia
SLE	Kidney (except in TTP-like syndrome)	Microthrombi composed of fibrin, lesions related with LN (class IV ++ > III – V). Frequent immune complex deposits, leucocyte infiltration/Glomeruli, small arterioles ± arteries	TTP like: platelets thrombi Other cases: fibrin thrombi
DIC	Most affected organs (descending order): kidney, liver, lung, heart, pancreas, adrenal gland, and GIT	Diffuse multiorgan bleeding, hemorrhagic necrosis, fibrin microthrombi in small vessels, and thrombi in medium and large vessels. No vasculitis	Fibrin thrombi

CNS, central nervous system; EC, endothelial cells; GIT, gastrointestinal tractus; LN, lupus nephritis; vWF, von Willebrand factor.

### 3.1 Defining thrombotic microangiopathy: a pathologist’s perspective

**Figure 1:**
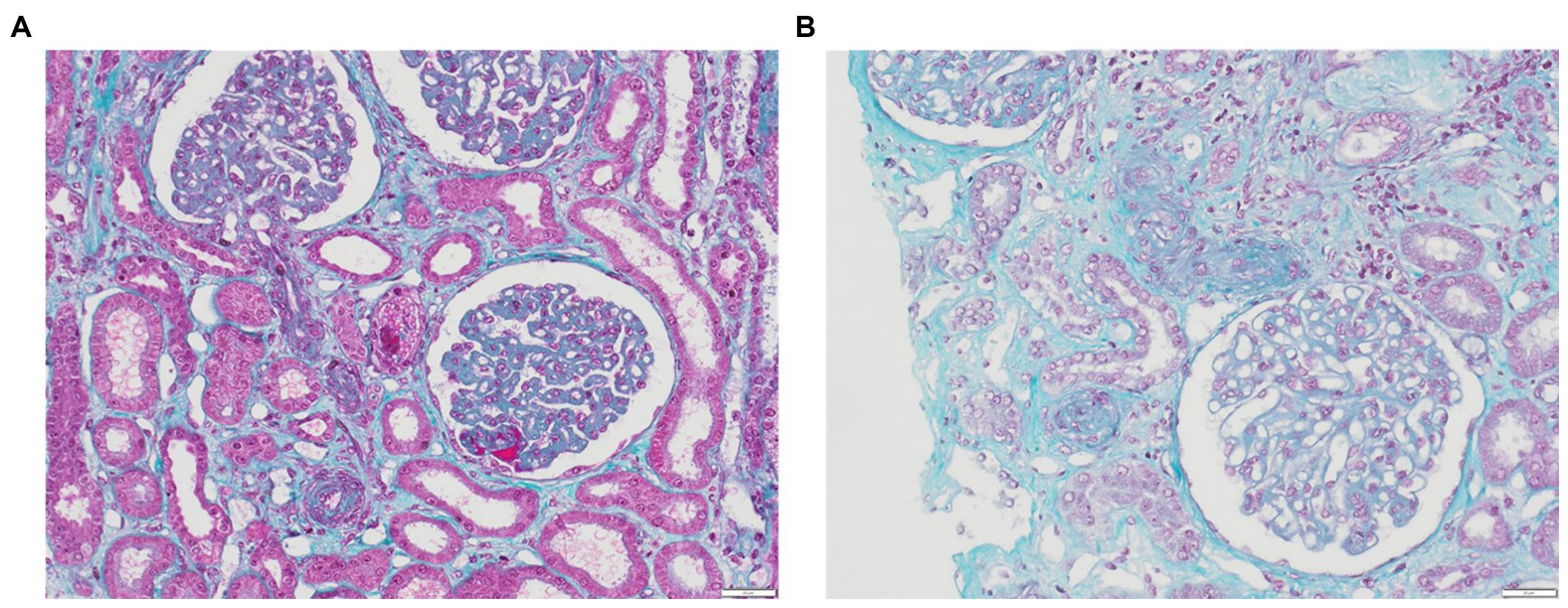
Renal TMA. **(A)** Glomeruli with GBM thickening due to subendothelial expansion, fibrin thrombi corresponding to acute TMA with swelling of EC in glomeruli and arterioles (trichrome stain, x20); **(B)** Acute TMA with arteriolar occlusions due to intimal edema and thrombosis (trichrome stain, x20).

TMA lesions are traditionally defined as being confined to microvessels, including arterioles, capillaries, small arteries, with a diameter cutoff of <200 μm (3). When assessing capillary lesions under light microscopy, endothelial swelling is the most prevalent, followed by subendothelial widening and capillary wall thickening. Among arteriolar lesions, fibrinoid necrosis, mucoid intimal thickening and luminal thrombi are reported (Figures 1A,B). Intraluminal thrombi usually comprise fibrins and platelets, although their composition can vary according to the pattern of TMA (Table 2) (28, 44, 56, 58, 99–102). While endothelial changes are consistently evident, thrombi may not always be visualized, especially in instances of early or mild disease. Downstream from the thrombi, necrotic lesions can be observed, resulting from the ischemic process. Consequently, acute tubular necrosis lesions are frequently reported in association with renal TMA lesions in cases of HUS (103), and liver enzymes are elevated in women with HELLP syndrome due to microangiopathy with sinusoidal obstruction, leading to hepatocyte necrosis (44, 100). Immunofluorescence typically reveals fibrinogen deposits localized to thrombi and also, in mesangium, in glomerular capillary walls and in afferent arterioles. Nonspecific entrapment of IgM and complement components is usually observed in glomeruli and affected vessels in around 50% of specimens, primarily in glomerular capillary walls, mesangium, and afferent arterioles. Staining for IgG and IgA consistently yields negative results (104, 105).

None of these lesions are specific, and the histological diagnosis of TMA remains complex, mainly due to the absence of established criteria. Furthermore, the potential overlap between acute (defined as occurring within 2 months from the initial presentation) and chronic features further complicates the histological diagnosis. Approaches to standardized analysis are beginning to be proposed, as evidenced recently in cases of TMA in renal transplant recipients, where they incorporate histological, clinical, and biological data. In this specific context, fibrin thrombi in arterioles and/or glomerular capillaries (100%), arteriolar intimal edema/mucoid changes (95%), and mesangiolysis (82%) demonstrated the highest level of consensus among the panelists (106).

### 3.2 Contribution of renal pathology in pregnancy related-TMAs

The literature primarily provides renal pathology data for pregnancy-related TMAs, possibly due to the frequency of postpartum AKI and the feasibility of renal biopsies. Although there are some histological descriptions of organ involvement beyond the kidney (detailed in Table 2), most of these descriptions originate from post-mortem examinations (99–102), possibly introducing bias by representing the most severe clinical cases, which could overestimate the prevalence of diffuse TMA lesions.

The kidney serves as the principal target of TMA in conditions such as PE, HUS, and CAPS, suggesting a specific vulnerability of this organ to TMA lesion development. While various hypotheses and emerging data, notably concerning an elevated renal susceptibility to complement activation, have been proposed, our understanding of this renal vulnerability remains constrained (107, 108). As mentioned previously, renal TMA is less common in HELLP syndrome, with predominant tubular lesions reported in series associated with AKI (66). The presence of fibrin deposition in hepatic sinusoids with periportal hemorrhage and hepatocyte necrosis is well-documented in post-mortem examinations of individuals with PE associated with HELLP syndrome. In contrast, these lesions are observed in liver needle biopsies in only 25% of eclampsia patients and are exceptionally rare in PE patients (44). These findings support the hypothesis of a clinical-pathological correlation between the extent of morphological changes and the severity of presentation, as well as adverse outcomes.

There is no documented correlation between the pathological features observed in renal biopsies and the underlying etiology of TMA. In non-gravid contexts, it has been suggested that the presence of isolated intimal mucoid edema without glomerular fibrin thrombi reduces the likelihood of CM-TMA in the setting of TMA associated with hypertensive emergencies (109); however, this remains a subject of controversy (103, 110). Additionally, some data suggest that renal arteriolar involvement is more widespread in aHUS and TTP, while glomerular involvement appears to be more prevalent in STEC-HUS (105). Nonetheless, it is important to note that the coexistence of these lesions is relatively common and has not been definitively linked to a specific underlying pathophysiological mechanism.

In gravid contexts, PE results in notable glomerular changes, termed “endotheliosis”, characterized by glomerular enlargement and reduced capillary blood flow due to endothelial swelling. This substantial reduction in endothelial cell fenestrations has been also observed in others capillary networks, including, choroid plexus, and hepatic sinusoids (104). This phenomenon is closely associated with sFlt-1, which reduces circulation of vascular endothelial growth factor (VEGF), impacting placentation and endothelial cell function, especially in fenestrated endothelium (40, 111). However, this specific lesion is not unique to PE and has been observed in various other conditions, including placental abruption, parvovirus B19 infection, COVID-19, POEMS syndrome, and even uncomplicated pregnancies (112). The presence of arteriolar or arterial lesions should prompt consideration of underlying conditions, like hypertensive vasculopathy, or alternative diagnoses, such as CM-HUS (105). *Post partum* biopsies can provide information about healing process or progression. For example, in PE, the endothelial swelling, IgM and fibrin deposits typically resolve within 2 weeks of delivery. The subendothelial irregularities in the glomerular basement membrane may persist for months (105). Renal biopsies can also help in lupus patients due to potential clinical similarities between LN flares and PE (113, 114). In APL cases, the presence of acute TMA in glomerular and arteriolar lesions alongside chronic vascular changes aids in diagnosis (115).

### 3.3 Contribution of placenta pathology in pregnancy related-TMAs

**Figure 2:**
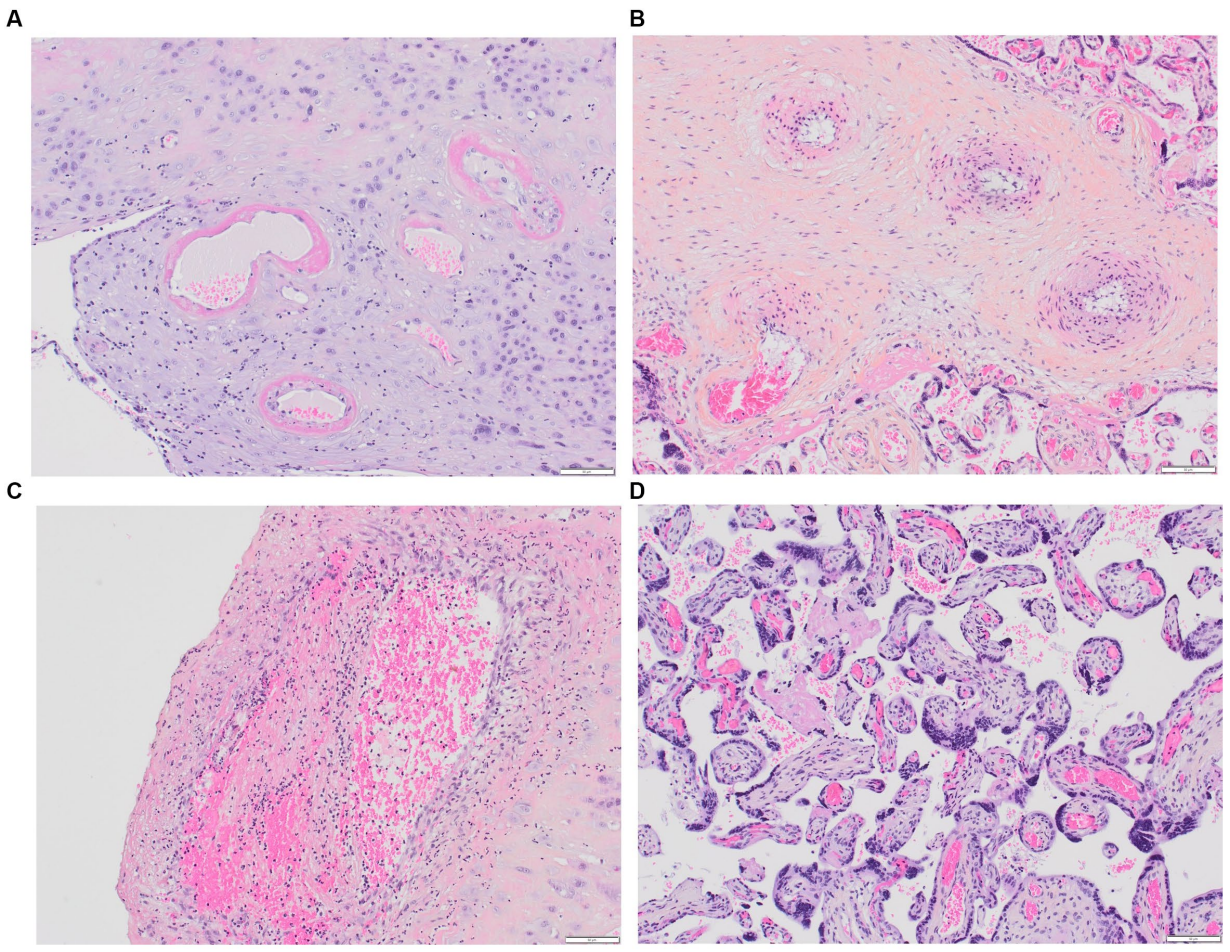
Placental MVM. **(A)** Maternal spiral arteries with acute atherosis and narrowed arteriolar lumens due to fibrinoid necrosis; **(B)** Recent maternal spiral artery thrombosis; **(C)** A section of free membranes showing decidual hypertrophic arteriopathy and residual smooth muscle; **(D)** This 23-week-gestation placenta exhibits accelerated villous maturation with small hypermature villi, increased syncytial knots, and intervillous fibrin.

In 2016, the Amsterdam Placental Workshop Group provided a consensus statement on placental lesion sampling and definitions (116). Presently, the accepted term is “maternal vascular malperfusion” (MVM), which encompasses a range of placental pathologic findings, including macroscopic features (fetal and placenta weights, infarcts, retroplacental hemorrhage) and microscopic findings (maternal decidual arteriopathy, distal villous hypoplasia, and accelerated villous maturation), Figures 2A–D. Maternal decidual arteriopathy comprises acute atherosis of decidual arteries, mural hypertrophy of arterioles within the placental membranes, abnormal persistence of mural smooth muscle within arteries at the placental basal plate, and the persistence of intramural endovascular trophoblast. The specific causes of MVM remain unclear, with potential factors including immunological and inflammatory elements, as well as pre-existing maternal susceptibility to microvascular dysfunction (117). MVM is described in PE and HELLP syndrome, and it also appears in instances of second-trimester spontaneous abortion, fetal demise, abruptio placentae, small for gestational age, preterm labor, preterm prelabor rupture of membranes, and maternal autoimmune diseases (117, 118).

Most of the placental histological data is centered around PE. Distinct lesions are predominant according the timing of PE during gestation, suggesting potential differences in underlying pathophysiological factors. Early-onset PE is characterized by more severe maternal MVM lesions, both macroscopic and microscopic (119). A positive correlation has been reported between the presence of decidual vasculopathy in vessels and adverse maternal and fetal outcomes (i.e., higher diastolic blood pressure, increased urinary protein levels, shorter gestational age, and lower birth weight) (112). In contrast, late-onset PE placentas show normal or excessive growth, milder microscopic features like accelerated villous maturation and hypertrophic arteriopathy (119), and significantly fewer cases of failed spiral artery remodeling, acute decidual arteriopathy, and infarcts compared to early-onset PE placentas (120). Mural hypertrophy of placental arterioles was linked to the development of stage 2 hypertension during follow-up (121), emphasizing the impact of preexisting cardiovascular risk factors (e.g., diabetes, chronic hypertension, obesity) in late-onset PE pregnancies. A limited number of studies have compared placental histopathological features in severe PE cases with and without HELLP syndrome (122–124). Notably, Weiner et al., in their analysis of 287 placentas, reported that vascular and villous lesions indicative of maternal malperfusion were independently linked to HELLP syndrome (124).

Information regarding other pregnancy-related TMAs is limited. In a cohort of 91 pregnancies involving 47 TTP-patients, Scully et al. examined placental histology from 15 deliveries. Interestingly, untreated congenital cases exhibited distal villous hypoplasia, widespread placental ischemia with infarcts of various ages, and acute atherosis, while treated congenital cases showed normal placental features (27). Similar results were reported in a recent study involving 28 placentas from congenital TTP cases (125), emphasizing also the direct role of ADAMTS-13 deficiency in placental findings. Moreover, some research groups have examined placental deposits of complement activation products, which have been identified in conditions such as PE, HELLP syndrome, lupus, and APS, without conclusive discriminatory features among these pathologies at present (126–128).

The data is still quite limited, and a more systematic analysis of placentas from patients being treated for TMAs could provide valuable insights, particularly when combined with the recently suggested use of electron microscopy to study placental pathologies during pregnancy (129).

## 4 Endothelium in pregnancy

Endothelial cells play a pivotal role in the pathophysiology of TMA (18, 130), implying that the specific occurrence of these events during pregnancy and the postpartum period is, to some extent, associated with microvascular alterations characteristic of pregnancy. Investigating the intricate mechanisms contributing to this increased susceptibility is imperative for enhancing our understanding of the pathogenesis of pregnancy-associated TMA and, consequently, for developing effective preventive and therapeutic approaches to mitigate its consequences.

### 4.1 Microvasculature heterogeneity, specificities of premenopausal women’s endothelium

In contrast to large vessels with their three tunics, capillaries, the primary site for TMA with arterioles, consist of a thin endothelium, basement membrane, subendothelial connective tissue, and a few pericytes, with diameters ranging from 4 to 10 μm and an endothelial wall of about 0.5 μm. They operate at low intravascular pressures (0–25 mmHg, up to 50 mmHg in kidney glomeruli) and slow flow rates (<1 mm/s), facilitating efficient plasma and tissue exchanges (131).

The phenotype of microvascular endothelial cells varies from one organ to another to fulfill specific functions and respond to the different needs of various tissues (132–134). As early as 2003, the spatial heterogeneity among endothelial cells was supported by distinct transcriptomic profiles (135) and differential expression of protein surface markers (136, 137). Most recently, the advent of single-cell RNA sequencing technologies has revolutionized our understanding of endothelial diversity, revealing significant heterogeneity within the confines of a single organ or a blood vessel type, in both animal models and humans (138–140). The endothelium is a dynamic tissue with plasticity properties, making it also subject to temporal heterogeneity (141). Endothelial cells react and respond to various biomechanical and biochemical stimuli that differ between organs, such as hormones, as discussed below, contributing to gender-driven endothelial heterogeneity. Additionally, site-specific epigenetic modifications play a significant role in generating endothelial heterogeneity (142, 143).

Significant sex-based differences in endothelial cells have been documented (144–146), emphasizing the heightened endothelial protection in women of childbearing age, which is associated with a lower cardiovascular risk compared to males within the corresponding age group or postmenopausal women. The studies have primarily focused on exploring the involvement and interactions of circulating sex steroid hormones, their receptors, and sex steroid-independent mechanisms. Notably, estrogen receptor E2-mediated vascular benefits encompass inhibition of lipoprotein oxidation, reduction of atherosclerotic lesions, modulation of blood coagulation, inhibition of collagen accumulation in vessels, and adjustment of vascular tone (144, 147). Studies conducted in animal models involving ovariectomy and hormone supplementation therapy have contributed to the understanding of sex hormone-dependent effects, particularly those of estrogen, on endothelial function, including changes in endothelium-dependent dilation, and the respective roles of nitric oxide (NO) and endothelium-derived hyperpolarizing factor (EDHF). These investigations have predominantly concentrated on medium and large arterial vessels (e.g., aorta, carotid, mesenteric arteries) (148–150). For instance, small arteries from omental tissue in premenopausal women demonstrate increased sensitivity to bradykinin (151); premenopausal women have shown increased levels of progenitor ECs in comparison to men, alongside enhanced NO production essential for vascular homeostasis (152); the profile of endothelial-derived factors in childbearing women is associated with a less pronounced pro-oxidant environment, characterized by reduced levels of vascular reactive oxygen species and other oxidative stress markers (153, 154). Additionally, levels of endothelin-1, which play a role in cardiovascular and renal pathology, are generally higher in men than in women (155); plasma levels of VEGF have been found to be elevated in males compared to females and have shown a positive association with the advancement of atherosclerosis (156).

Collectively, these studies underscore the heightened endothelial protection observed in women of childbearing age, elucidating their diminished susceptibility to cardiovascular events. They also illustrate the importance of incorporating both genders into experimental protocols, also including *ex vivo* and *in vitro* studies. Nonetheless, questions and controversies debates, especially concerning the strict favorable vascular impacts of estrogen and its E2 receptor (157).

Furthermore, as previously emphasized, research on vascular sex differences has predominantly focused on macrovessels, while data on the microcirculation are limited (158). Some studies have demonstrated sex differences in the permeability or inflammation-induced disruption of the blood–brain barrier (159, 160), and circulating levels of products derived from the glycocalyx (e.g., syndecan-1, heparan sulfate, and hyaluronan) (161, 162). More answers may potentially come from single-cell RNA-seq analyses. Already, a single-cell RNA-seq analysis of mouse endothelial cells has further confirmed the presence of sex-specific differences in endothelial cells across various mouse tissues. Paik et al. found that markers of tissue-specific endothelial cells, enriched pathways, and endothelial subpopulations differed between male and female mice, particularly in the brain, heart, and lung. The Lars2 gene, encoding mitochondrial leucyl-tRNA synthetase, was notably more enriched in male than in female endothelial cells (163). While this comprehensive approach has yet to be undertaken in pregnant individuals or TMA patients, specific endothelial attributes associated with pregnancy have been recognized and may contribute to the heightened thrombogenicity of the gravid endothelium.

### 4.2 Specificities of the gravid endothelium

**Figure 3:**
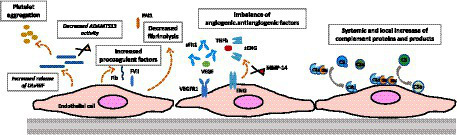
Gravid endothelium—pregnancy-induced adaptations. This figure highlights pregnancy-induced changes in the endothelium, emphasizing adaptations in complement activation, prothrombotic profile, and angiogenic state. These modifications underscore the complexity of the gravid endothelium, crucial for successful pregnancy outcomes.

Vascular adaptations during pregnancy, even in healthy pregnancies, are associated with significant modifications in the characteristics and function of endothelial cells. For instance, glomerular capillary endotheliosis, originally associated to PE, has been detected in patients with gestational hypertension without proteinuria (> 1+ in 8/8, and > 2+ in 4/8 cases) and, as cited above, in women with healthy pregnancies (>1+ in 5/12, and > 2+ in 1/5 cases) based on renal biopsies conducted between 27 and 39 weeks of gestation (112). These findings suggest a continuum of endothelial changes that range from normal pregnancy to the development of PE. Multiple vascular changes take place during pregnancy, potentially heightening the vulnerability to thrombosis, and our primary focus in this context is on the specific factors involved in the pathophysiology of TMAs (Figure 3).

#### 4.2.1 Complement activation

Complement activation via the three pathways - classical, lectin, and alternative - has been observed during normal pregnancy. It has been suggested that this activation is physiological, serving as a regulatory mechanism to facilitate the clearance of fetal placental debris and protect both the mother and developing fetus from pathogens (4, 82, 164, 165). The complement system plays a significant role in various stages of pregnancy, such as implantation, placentation, fetal development, and parturition (164, 166). This likely contributes to the variations in plasma levels and placental deposition/expression of complement components during this period.

Derzsy et al. reported significantly elevated levels of various complement proteins and activation products, including C4d (~2.75-fold), C3a (~8.8-fold), soluble C5b9 (~1.8-fold), C3 (~1.2-fold), and C9 (~1.3-fold), while C1-inhibitor concentrations were significantly lower (~0.8-fold), in healthy pregnant women sampled between 36 to 67 weeks of gestation (*n* = 60) compared with healthy non-pregnant women (*n* = 59). There was no significant modification in the alternative pathway fragment Bb (76). More recently, a study analyzing sequentially collected 733 plasma samples from 362 women with a normal pregnancy and 65 samples from non-pregnant women confirmed an increase in the levels of C3, C4, factor B, and mannose binding lectin, while the levels of C1q remained unchanged. The levels of C3a, and to a lesser extent, C5a, remained elevated throughout pregnancy, while soluble C5b-9 levels remained unchanged, suggesting that excessive complement activation does not progress beyond the C3 and C5 levels in normal pregnancy (79). Interestingly, the levels of factor H (FH), the principal regulator of the alternative pathway, whose default has been shown to be associated with a risk of developing CM-HUS, increased significantly, gradually up to 28 weeks of gestational age, and then remained stable (76, 79).

Several studies have shed light on the presence of complement activation product deposits and the expression of regulatory proteins within placentas from healthy pregnancy (164, 166). Notably, deposits of C3d and C9 have been reported in trophoblast basement membranes, along with staining for C1q, C3d, C4, C6, and C9 in normal uteroplacental spiral arteries (167–169). The presence of C5b-9 has also been documented in the normal term placenta, specifically within the decidua and the stroma of chorionic villi (170). These findings suggest that the placenta functions as a localized site of complement activation. Interestingly, it has been observed that human trophoblasts synthesize complement molecules such as C4, C3, and the late complement components (C6, C7, C8, C9) (171). Considering this complement activity, a notable presence of complement regulatory proteins has been observed within trophoblasts. Lokki et al. identified the presence of C4BP in syncytial bodies or regions with damaged syncytiotrophoblasts, as well as FH within tissue stroma (168), and trophoblasts have been recognized for their synthesis of FH (172). Some studies have also reported an elevated expression of membrane complement proteins (CD46, CD55, CD59) on extravillous trophoblasts during healthy pregnancies (164, 169).

These findings underscore the substantial involvement of complement activity in placental homeostasis. It is noteworthy that the current knowledge does not offer a concise rationale for the increased susceptibility to CM-HUS in the postpartum period. One hypothesis suggests that fetal-derived complement regulatory factors might exert a compensatory effect that diminishes after childbirth.

#### 4.2.2 Prothrombotic profile

The heightened thrombotic risk during pregnancy is well-documented. When compared to non-pregnant women, there is a three to fourfold increase in the risk of arterial thromboembolism [e.g., stroke (173), myocardial infarction (174)], and a four to fivefold increase in the risk of venous thromboembolism (175). After childbirth, the risk of venous thromboembolism becomes even more significant, a twenty-fold increase. The primary cause of the heightened risk is hypercoagulability, which is believed to have developed as an evolutionary adaptation to safeguard women against the bleeding complications associated with miscarriage and childbirth. Hemostatic changes that lead to this hypercoagulable state during pregnancy encompass all facets of hemostasis, including primary hemostasis, coagulation, and fibrinolytic capacity. Numerous factors, including circulating factors, platelets, endothelial cells, and others, play critical roles in this process, particularly at the maternal-fetal interface. The Table 3 reports the per-pregnancy dynamic of important endothelial-derived factors.

**Table 3:** Main derived-endothelial factors involved in hemostasis and their dynamics during normal pregnancy.

	Prothrombotic factors	Function	Dynamic in healthy pregnancy	Antithrombotic factors	Function	Dynamic in healthy pregnancy
Primary hemostasis	*vWF*	Platelets aggregation, FVIII transport	**↑**	*ADAMTS-13*	Cleavage of vWF multimers	**↓**
	*TXA2*	Platelets aggregation, vasoconstriction	↓	*NO*	Inhibition of platelets activation, vasorelaxation, anti-inflammatory properties	↑
	*PAF*	Platelets aggregation, vasoconstriction	No data	*PGI2*	Inhibition of platelets activation, vasorelaxation	↑
Coagulation	*Tissue factor*	Binding to FVII, initiation of blood coagulation (extrinsic pathway)	**↑**	*TPFI/TFPI-2*	Inhibition of coagulation by binding to TF, factors Va, and Xa	→/↑
	*PARs*	Thrombin receptors	No data	*TM*	Formation of a thrombin complex enhancing protein C affinity	↑
				*Heparan-sulfate*	Binding to antithrombin	Few data, glycocalyx thinning
Fibrinolysis	*PAI-1*	Fibrinolysis inhibitor	**↑**	*t-PA*	Fibrinolysis activator	↑

NO, nitric oxide; PAI-1, plasminogen activator inhibitor type 1; PAF, platelet activating factor; PARs, protease activated receptor; PGI2, prostacyclin I2; t-PA, tissue plasminogen activator; TFPI, tissue factor pathway inhibitor; TM, thrombomodulin; TXA2, thromboxane A2; vWF, von Willebrand factor.

An increase in platelet aggregation is documented during a healthy pregnancy (176, 177), although it is not consistently observed in all studies (178). These potential alterations in platelet aggregation coincide with a substantial two- to fourfold increase in von Willebrand factor levels, originating from endothelial Weibel-Palade bodies (179, 180). Concurrently, ADAMTS13 activity exhibits a noteworthy decline of 20–50%, persisting from the second trimester through delivery and into the early postpartum period (180, 181). This decline in ADAMTS13 activity is attributed to consumption resulting from elevated levels of von Willebrand factor and hormonal fluctuations, although it typically remains above 20% (179, 181). These physiological changes can exacerbate the low ADAMTS13 levels in individuals with congenital TTP to a critical level resulting in overt presentation of TTP. Other endothelial-derived factors contribute to the maintenance of hemostatic balance during a normal pregnancy. In comparison to non-pregnant controls, the ratio of prostacyclin (PGI2) to thromboxane A2 (TXA2) metabolites gradually increases from the second trimester onwards (182), and NO concentrations exhibit a significant elevation throughout pregnancy (183). It is noteworthy that these patterns diverge in the context of PE, particularly in severe cases (183–185).

Significant changes in blood coagulation factors during a healthy pregnancy are also documented and primarily attributed to hormonal fluctuations, particularly the increasing levels of estrogen as pregnancy progresses (147). Notably, plasmatic levels of factors VII, VIII, X, XII, and fibrinogen increase substantially, especially in the third trimester (33, 186–188). Furthermore, while endothelial cells typically express minimal tissue factor (TF) under physiological conditions (189, 190), maternal decidua and the placental syncytiotrophoblasts exhibit elevated levels of TF with a strong affinity for factor VII, initiating thrombin generation. During extravascular trophoblast invasion of the decidua, thrombin produced via TF expressed by decidual cells serves to prevent hemorrhage during the initial capillary breach and subsequent invasion and remodeling of spiral arteries and arterioles (191). While free protein S levels decrease by approximately 55%, other anticoagulant factors, such as thrombomodulin and tissue factor pathway inhibitor-2, exhibit increased expression in the placental vascular endothelium with advancing gestational age (33, 192, 193).

Regarding fibrinolysis, while levels of tissue plasminogen activator also rise during a normal pregnancy (194), concomitantly elevated levels of plasminogen activator inhibitor-1 (PAI-1) from endothelial cells and plasminogen activator inhibitor-2 (PAI-2) from the placenta, on the contrary, lead to a decrease in fibrinolytic capacity (188).

All these changes in hemostasis underscore the critical importance of achieving a precise balance for normal placental function and favorable pregnancy outcomes. While intricacies exist even at the level of the maternal-fetal-placental vascular beds (for instance, the TF/TFPI ratio is markedly elevated in syncytiotrophoblasts, whereas it is lower in HUVECs, implying a more procoagulant tendency of syncytiotrophoblasts) (193), the progression of human gestation involves a transition of endothelial cells toward a procoagulant state. This prothrombotic profile may endure until approximately 8 weeks postpartum.

#### 4.2.3 Others actors

Additional modifications, which have the potential to impact susceptibility to TMA, are concomitant with a normal pregnancy. Placental microvascular endothelial cells undergo noteworthy fluctuations in their angiogenic state throughout pregnancy. The first and second trimesters are characterized by a predominance of angiogenic activity, facilitating rapid placental expansion. In contrast, as pregnancy progresses toward full term, there is a transition to a prevailing angiostatic condition, coinciding with placental growth arrest. Placental cells inherently possess the capacity for angiogenic regulation, engage in intricate interactions with perivascular cells, and demonstrate the ability to swiftly adapt and remodel in accordance with the specific demands of pregnancy. A longitudinal study reported the dynamic patterns of different angiogenesis factors of interest. sFlt1 levels remained relatively stable until weeks 29–30, after which they significantly increased, peaking at week 40 with a notable weekly increase of 643 pg./mL. In contrast, PlGF levels increased gradually up to weeks 29–30, followed by a steady decline of 14 pg./mL per week until week 40. The sFlt1:PlGF ratio decreased from weeks 9–12, remained consistently low from weeks 19–20 to 37–38, and then increased again toward weeks 39–40. Additionally, VEGF-A was detectable in only 8% of the samples during pregnancy but increased to 64% in the postpartum period (195). Therefore, the effective functioning of placental microvascular endothelial cells holds paramount importance for the successful progression of pregnancy (196).

Even more limited data exist regarding changes in the glycocalyx throughout pregnancy. Changes in glycocalyx thickness, along with reported shedding, have been documented and could potentially contribute to observed alterations in fluid balance during pregnancy (158, 197). Limited data is available regarding changes in glycocalyx composition, especially concerning heparan sulfates. This aspect is of particular interest due to their anticoagulant action through binding to antithrombin III and their role as inhibitors of complement activation via FH binding. Interestingly, a recent transcriptomic analysis using microarrays revealed that among the 34 expressed proteoglycans in the placenta, syndecan-1 (SDC1) production is significantly the highest, and SDC1 is the most upregulated gene during the differentiation of trophoblast into syncytiotrophoblast (198). This suggests the existence of a pregnancy-specific glycocalyx that distinguishes itself from the glycocalyx of both adult and fetal endothelium and whose balance could contribute to the successful progression of pregnancy (199).

### 4.3 Overcoming endothelial regulation: from physiology to pathology in pregnancy

Therefore, the “gravid endothelium” is inherently more prone to activation, contributing to the favorable progression of pregnancy. The transition to a pathological state with the development of TMA lesions requires additional factors, as described in the concept of a “multiple-hits disease” (18, 200). These additional factors can be either genetic and/or acquired, and act to heighten susceptibility and/or induce endothelial stress. Their cumulative impact (with varying significance depending on the specific factor) will ultimately lead to an exceeding of the regulatory capacities involved in endothelial homeostasis.

Genome-wide association studies have not been conducted in women experiencing pregnancy-related TMAs. Such investigations have primarily been carried out in the context of PE, often without a clear differentiation of patient phenotypes. While more than fifty candidate genes have been associated with PE, no universally accepted susceptibility genes have emerged (41, 201, 202), possibly due to the polygenic nature of PE. In a study of 244,564 related women, Cnattingius et al. determined that PE has a heritability of over 50%, with 35% linked to maternal genetics, 20% to fetal genetics, and 13% to a ‘couple’ effect (203). Mutations and/or variants have been reported in the context of PE or HELLP syndrome, related to genes involved in angiogenesis, blood pressure regulation, coagulation, or complement activation (40, 202, 204, 205), underscoring the close pathophysiological connections with TMA. For instance, mutations or variations in Factor H, C3, or CD46 have been identified in cohorts of women with PE (206, 207). These findings indicate that a multifaceted interplay of susceptibility factors is likely, potentially resulting in distinct patterns of preeclampsia. Supporting this concept, Schuster et al. documented groups of patients exhibiting common gene and protein networks linked to severe preeclampsia (208).

The extensive range of environmental factors capable of increasing endothelial susceptibility cannot all be comprehensively reviewed here. In an illustrative example, placental hypoxia is worth mentioning to illustrate the interplay of factors involved in TMA. The placenta is a physiological immunological niche, especially under the influence of physiological hypoxia that contributes to immunological regulation (209). However, placental hypoxia has others consequences as the generation of oxidative stress and trophoblastic secretion of sFLT-1, promoting antiangiogenic activities and endothelial injury (210), alteration in placental metabolism favoring interruption of the glycocalyx susceptible to contribute to localized complement activation (199, 211, 212). Thus, a pathological placental hypoxia, as observed in the case of PE, represents a risk situation for TMA.

## 5 Conclusion

The susceptibility of the gravid endothelium to instigate TMA represents a pivotal facet within the realm of vascular pathology. During pregnancy, intricate physiological adaptations occur within the vascular milieu, rendering the endothelial lining more vulnerable to disruptions in hemostasis. This heightened susceptibility is attributed to a multifaceted interplay of hormonal, immunological, and hemodynamic factors, culminating in an environment conducive to dysregulated endothelial activation and subsequent TMA development. Elucidating the nuanced mechanisms underlying this heightened propensity is essential for advancing our comprehension of pregnancy-associated TMA pathogenesis and, subsequently, devising effective preventive and therapeutic strategies to mitigate its impact.

## Author contributions

MF: Writing – original draft, Writing – review & editing. VG: Writing – original draft, Writing – review & editing. MS: Writing – original draft, Writing – review & editing. FP: Writing – review & editing. FF: Writing – original draft, Writing – review & editing.
